# Insertion sequences drive the emergence of a highly adapted human pathogen

**DOI:** 10.1099/mgen.0.000265

**Published:** 2019-04-04

**Authors:** Erwin Sentausa, Pauline Basso, Alice Berry, Annie Adrait, Gwendoline Bellement, Yohann Couté, Stephen Lory, Sylvie Elsen, Ina Attrée

**Affiliations:** ^1^​ Université Grenoble Alpes, CNRS ERL5261, INSERM U1036, CEA, Laboratory Biology of Cancer and Infection, Bacterial Pathogenesis and Cellular Responses, Biosciences and Biotechnology Institute of Grenoble, 38000 Grenoble, France; ^2^​ Université Grenoble Alpes, CEA, Inserm, BIG-BGE, 38000 Grenoble, France; ^3^​ Department of Microbiology and Immunobiology, Harvard Medical School, Boston, MA, USA; ^†^​ Present address: Evotec ID (Lyon) SAS, Marcy l’Étoile, France.; ^‡^​ Present address: Department of Microbiology and Immunology, University of California San Francisco (UCSF) School of Medicine, San Francisco, CA, USA.; ^§^​ Present address: Biozentrum, University of Basel, Basel, Switzerland.

**Keywords:** *Pseudomonas aeruginosa*, adaptation, antibiotic resistance, bacterial infection, multiomics

## Abstract

*Pseudomonas aeruginosa* is a highly adaptive opportunistic pathogen that can have serious health consequences in patients with lung disorders. Taxonomic outliers of *P. aeruginosa* of environmental origin have recently emerged as infectious for humans. Here, we present the first genome-wide analysis of an isolate that caused fatal haemorrhagic pneumonia. In two clones, CLJ1 and CLJ3, sequentially recovered from a patient with chronic pulmonary disease, insertion of a mobile genetic element into the *P. aeruginosa* chromosome affected major virulence-associated phenotypes and led to increased resistance to the antibiotics used to combat the infection. Comparative genome, proteome and transcriptome analyses revealed that this ISL3-family insertion sequence disrupted the genes for flagellar components, type IV pili, O-specific antigens, translesion polymerase and enzymes producing hydrogen cyanide. Seven-fold more insertions were detected in the later isolate, CLJ3, than in CLJ1, some of which modified strain susceptibility to antibiotics by disrupting the genes for the outer-membrane porin OprD and the regulator of β-lactamase expression AmpD. In the *Galleria mellonella* larvae model, the two strains displayed different levels of virulence, with CLJ1 being highly pathogenic. This study revealed insertion sequences to be major players in enhancing the pathogenic potential of a *P. aeruginosa* taxonomic outlier by modulating both its virulence and its resistance to antimicrobials, and explains how this bacterium adapts from the environment to a human host.

## Data Summary

All data are available. The whole-genome shotgun projects for CLJ1 and CLJ3 have been deposited at DDBJ/ENA/GenBank under accession numbers PVXJ00000000 and PZJI00000000, respectively. The raw sequencing data were deposited at NCBI’s Sequence Read Archive under accession numbers SRP170343 and SRP170410. The RNA-Seq datasets have been uploaded to GEO under identifiers GSE123106 and GSE123107. The MSy proteomics data were deposited with the ProteomeXchange Consortium via the PRIDE partner repository under dataset identifier PXD011105. These data can be accessed on https://www.ebi.ac.uk/pride/archive/. The RNA-Seq experiments were performed in accordance with the ENCODE guidelines and appropriately validated by RT-qPCR.

**Impact Statement**

Bacteria employ diverse strategies to adapt to changing environments, including the infectious niche in human hosts. *Pseudomonas aeruginosa* adapts to establish a chronic infection in cystic fibrosis patients through small nucleotide changes in pathoadaptive genes. In this study, we demonstrate that *P. aeruginosa* clonal outliers use another strategy to evade the host’s immune system and to deal with the antibiotics used to treat the infection. Using a genome-wide ‘multiomics’ approach, we discovered that a single mobile genetic element belonging to the ISL3 insertion sequence family altered (i) the structure of multiple surface macromolecules (flagella, pili, lipopolysaccharide), (ii) the levels of proteins responsible for antibiotic resistance, and (iii) the expression of some virulence factors. Our work thus reveals another powerful means used by bacteria to rapidly adapt to a human niche and deal with the threat posed by antimicrobials.

## Introduction

Emerging infectious diseases caused by multidrug-resistant bacteria represent a serious threat to human well-being and health. Over the last 40 years, several hundred novel pathologies caused by infectious agents have been reported [1]. Environmental bacteria can adapt to a human host by acquiring virulence traits through chromosomal rearrangements due to the insertion of mobile genetic elements, horizontal gene transfer or small local sequence changes, such as single nucleotide substitutions [2, 3].

The genus *Pseudomonas* is one of the largest groups of bacteria, found in diverse environments and capable of causing both plant and animal diseases. *Pseudomonas* species such as *P. aeruginosa*, *P. fluorescens* and *P. syringae* adversely impact human health and agriculture [4–6]. *P. aeruginosa* is a particularly successful opportunistic pathogen frequently found in humid environments associated with human activities. In hospital settings, infections caused by multidrug-resistant *P. aeruginosa* strains present a real danger for elderly individuals, patients treated with immunosuppressive therapies and users of invasive devices in intensive care units. In addition to acute infections, *P. aeruginosa* is a common cause of chronic wound infections, and is linked to long-lasting respiratory infections in patients with cystic fibrosis (CF) and chronic obstructive pulmonary disease (COPD). During chronic infections, the bacteria adjust to their particular host environment by adapting their metabolic pathways and synthesizing particular virulence-associated components [7]. Alterations occurring post-colonization include acquisition of loss-of-function mutations in genes of motility and antibiotic resistance, as well as aggressive factors responsible for acute virulence [8–12].

Recent massive whole-genome sequencing has allowed the classification of clinical and environmental *P. aeruginosa* strains into four well-defined clades [13, 14]. The different clades rely on different toxins to exert their pathogenic strategies. The two most populated clades inject toxins, also referred to as effectors, ExoS, ExoT, ExoY and ExoU, directly into the host cell cytoplasm using a type-III secretion system (T3SS) [15]. The third and fourth clades are occupied by strains lacking all the genes encoding the effectors and the components of the T3SS. The first fully sequenced taxonomic outlier, PA7, was found to be multi-drug resistant and non-virulent in a mouse model of acute lung infection [16, 17]. Other PA7-related strains were mainly of environmental origin [18, 19], but were occasionally associated with acute (wounds and urinary tract) and chronic (CF and COPD) human infections [20–22]. Some recently emerged highly virulent clones from those clades secrete the pore-forming toxin Exolysin, ExlA, that was found to be responsible for bacterial cytotoxicity [16, 21–24]. The most pathogenic *exlA* ^+^ *P. aeruginosa* strain described to date is CLJ1, which was isolated at the University Hospital in Grenoble, France, from a COPD patient with haemorrhagic pneumonia [16]. Experimentally infected mice with CLJ1 had extensive damage to their lung endothelial monolayers, and bacteria had transmigrated into the blood and disseminated into secondary organs, all without being detected by the host’s immune system. This clinical scenario differs greatly from the consequences observed with the T3SS^+^ strain PAO1 [16, 25].

To gain insight into the molecular determinants of pathogenesis expressed by Exolysin-producing *P. aeruginosa* taxonomic outliers, and to assess the extent of evolutionary adaptation during the course of infection, we performed a comprehensive comparative genome-wide study of two clonal variants, CLJ1 and CLJ3, isolated from the same patient at different time-points during hospitalization. The data gathered demonstrated that mobile genetic elements belonging to the ISL3 insertion sequence (IS) family, originally found in soil bacteria *P. stutzeri* and *P. putida*, shaped the virulence traits and strategies used by these strains to colonize their human host, escape from the immune system and resist antibiotic treatment.

## Methods

### Bacterial strains and culture conditions

The *P. aeruginosa* strains used in this study were CLJ1 and CLJ3 [16]. Bacteria were grown at 37 °C in liquid Lysogeny broth (LB) medium (per litre: 10 g Bacto tryptone, 5 g yeast extract, 10 g NaCl) with shaking until the cultures reached an optical density of 1.0 (or other value where indicated) at 600 nm (OD_600_). To assess levels of cyclic-di-GMP (c-di-GMP), pUCP22-p*cdrA-gfp*(ASV)^c^ was introduced into CLJ1 and CLJ3 strains by electroporation, as previously described [26], and transformed bacteria were selected on LB agar plates containing carbenicillin at 200 µg ml^−1^.

### Genome analysis

**Fig. 1.**
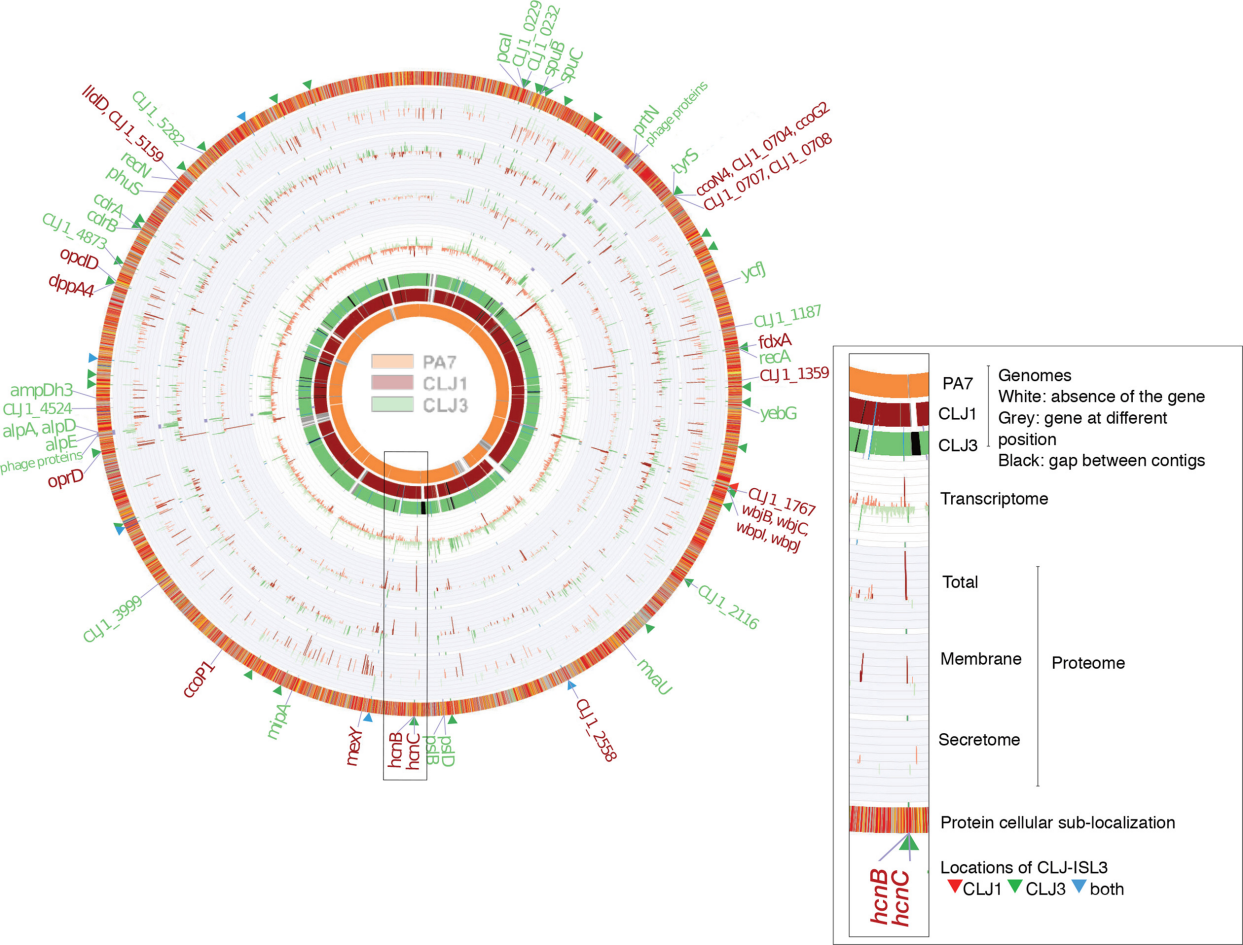
Comparison of the CLJ1 and CLJ3 genomes, transcriptomes and proteomes. The overview of the two genomes, compared to the PA7 strain reference genome, is shown on the left. The image on the right is an enlarged genomic segment at the *hcn* locus presenting a more detailed description of the data. The three genome rings in the figure represent the pangenomes for the different strains. Grey blocks in the PA7 ring indicate that the homologue of the CLJ1 (and CLJ3) gene in that position exists elsewhere in the PA7 genome. Genes were ordered based on the ordering of genes in the PA7 genome, remaining genes were ordered according to their positions in the CLJ1 contig, or when not present in either PA7 or CLJ1, the ordering of genes in CLJ3 contigs. The red bars on charts indicate genes or proteins that were more abundantly expressed in CLJ1, whereas the green bars indicate higher expression levels in CLJ3; darker tones indicate statistically significant differences in expression levels between the two strains (false discovery rate<0.05). The labels on the outermost ring highlight genes that were differentially expressed in both RNA sequencing (RNA-Seq) and at least one of the proteomics datasets. The protein subcellular localization (outermost ring) was coloured according to the coding used in the Pseudomonas Genome Database [30]. CLJ-ISL3 insertions are indicated by red, green and blue triangles, depending on whether they were present in CLJ1, CLJ3 or both strains, respectively.

Details of sequencing, assembly, annotation and comparison can be found in the Supplementary Materials and Methods. The assembly and annotation statistics for the CLJ1 and CLJ3 genomes are presented in Table S1 (available in the online version of this article). Functional annotation was performed on the Rapid Annotations based on Subsystem Technology (RAST) Genome Annotation Server, version 2.0 [27], using the Classic RAST annotation scheme and the GLIMMER-3 gene caller. Annotations were manually curated based on annotations of orthologous genes in PA7 and PAO1 strains in the Pseudomonas Genome Database [28]. Circos 0.69-3 [29] was used to create the multiomic data visualizations for Figs 1 and S1.

### Identifying insertion sites for IS elements

Locations of CLJ-ISL3 insertions in the gaps between CLJ3 contigs were detected by checking for the inverted repeat sequences at contig ends, while taking the shared gene synteny between CLJ1, PA7 and PAO1 genomes into account. PanISa version 0.1.0 (https://github.com/bvalot/panISa) [30] was also used, with default parameters, to search for ISs in CLJ3 reads. Reads were mapped to the PA7 and CLJ1 genomes using the BWA-MEM algorithm from BWA version 0.7.15 [31].

### Transcriptome

The RNA for RNA-Seq was prepared as previously described [32] from bacterial cultures grown in duplicate in LB to an OD_600_ of 1. Briefly, total RNA was isolated using the Direct-zol RNA MiniPrep kit (Zymo). Cells were collected by centrifugation (8000 ***g***, 5 min) and the supernatant was discarded. TRI Reagent (700 µl) was added to the pellet, and bacteria were lysed by vigorous vortexing (2 min). After centrifugation at 13000 ***g*** for 1 min, the lysate was transferred to a clean tube containing an equal volume of 100 % ethanol. The ethanol mixture was transferred to a Zymo-Spin IIC Column placed in a collection tube and centrifuged. The column, to which RNAs were bound, was washed according to the manufacturer’s protocol. RNA was finally eluted with 40 µl nuclease-free water. rRNA was depleted using the Ribo-Zero magnetic kit (Epicentre). The Illumina libraries and sequencing were prepared according to standard procedures at the Biopolymer Facility, Harvard Medical School, Boston, USA. Data were analysed as described in the Supplementary Materials and Methods. The total number of reads mapped to the genes was incorporated into a tabular format and analysed as raw counts using the DESeq2 differential expression analysis pipeline. Differentially expressed genes between CLJ1 and CLJ3 were identified using a 5 % false discovery rate (FDR).

### MS-based quantitative proteomics analyses

Proteomics samples were prepared and analysed by nano-LC coupled to tandem MS (Ultimate 3000 coupled to an LTQ-Orbitrap Velos Pro; Thermo Scientific) as described previously [33] with slight modifications (see Supplementary Materials and Methods). Each fraction was verified by western blot for purity using appropriate antibodies. The protein content in the whole-cell, membrane and secretome fractions of CLJ1 and CLJ3 was analysed independently. Statistical analyses were performed using ProStaR [34]. In total, a list of 2852 quantified proteins was obtained. The MS proteomics data were deposited with the ProteomeXchange Consortium via the PRIDE [35] partner repository under dataset identifier PXD011105.

### Phenotypic analyses

HCN production was assessed on induction plates containing arginine (an HCN precursor), as previously described [22]. Briefly, a sheet impregnated with a Cu^2+^-containing reaction mixture was placed above agar plates seeded with bacteria. A white-to-blue colour transition indicates HCN production. The fluorescence-based reporter plasmid pUCP22-p*CdrA-gfp*(ASV)^c^ was used to monitor levels of c-di-GMP [36]. Bacteria carrying the plasmid were subcultured to an OD_600_ of 0.05 in a black 96-well plate with a clear bottom, and incubated at 37 °C under 60 r.p.m. in the Fluoroskan reader. Fluorescence was measured every 15 min for 6 h, and emission was monitored at 527 nm following excitation at 485 nm. Serum sensitivity was assessed by a protocol adapted from the literature [37]. Two different human sera provided by the French National blood service (EFS) were used in all experiments. Overnight cultures of CLJ1 and CLJ3 bacteria were pelleted at 3000 r.p.m. for 5 min and suspended in Hanks balanced salt solution (HBSS; Gibco) with 0.1 % gelatin, adjusted to 10^8^ c.f.u. ml^–1^. Bacteria (10^6^) were incubated in the presence of 10 % human serum in a final volume of 3 ml for 15 min or 30 min at 37 °C under gentle agitation. A negative control with heat-inactivated serum (56 °C for 30 min) was included. Numbers of c.f.u. were determined at 0, 15 and 30 min by serial-dilution and spreading on LB plates. Statistical significance was determined using a *t*-test.

### Infections of *Galleria mellonella* larvae

Calibrated larvae of the wax moth *Galleria mellonella* were purchased from Sud-Est Appats (http://www.sudestappats.fr). Healthy, uniformly white larvae measuring around 3 cm were selected for infection. The bacteria were grown to an OD_600_ of 1 and diluted in PBS to approximately 10^3^ bacteria ml^–1^. Insulin cartridges were sterilized before filling with bacterial solutions. Larvae were injected with 10 µl of bacterial suspensions using an insulin pen. The precise number of bacteria transferred in injections was determined by spotting 10 µl aliquots with the pen five times onto agar plates, and counting c.f.u. after growth at 37 °C for 16 h. Infected larvae were placed in Petri dishes and maintained at 37 °C. Dead larvae were counted over the period indicated. Twenty larvae were used per condition, and the experiment was performed twice.

### RT-qPCR

To quantify selected transcripts, total RNA from 2.0 ml cultures (OD_600_ of 1.0) was extracted with the TRIzol Plus RNA Purification Kit (Invitrogen) and treated with DNase I (Amplification Grade; Invitrogen). Real-time quantitative PCR (RT-qPCR) was performed as described [38] with minor modifications (see Supplementary Materials and Methods). Primer sequences were designed using Primer3Plus (http://www.bioinformatics.nl/cgi-bin/primer3plus/primer3plus.cgi/) and are listed in Table S2. Transcript quantifications are shown with sem.

### Statistical analyses

Statistical analyses were performed with ‘T-Test Calculator for 2 Independent Means’ (https://www.socscistatistics.com/tests/studentttest), or a log-rank test. The threshold for statistical significance was set at *P*<0.05.

## Results

### Analysis of the CLJ1 genome and its regions of genomic plasticity

CLJ1, an antibiotic-sensitive *P. aeruginosa* strain, was isolated from a patient with necrotizing haemorrhagic pneumonia. Twelve days after initiating antibiotic therapy, the patient’s condition worsened and a multidrug-resistant clonal variant, CLJ3, was isolated [16]. CLJ1 shares the main genomic features with the first fully sequenced antibiotic-resistant *P. aeruginosa* taxonomic outlier, PA7 [16, 17]. In particular, CLJ1 lacks the entire locus encoding the T3SS machinery and the genes encoding all known T3SS effectors; however, it carries the determinant for the two-partner secretion pore-forming toxin, Exolysin. To initiate genome-wide studies into the mechanisms conferring the specific CLJ1 phenotypes, we sequenced the genome of this strain and compared it to PA7. As expected, most core genes were shared by the two strains (Figs 1 and S1). However, differences in the content and distribution of several variable regions, or ‘regions of genomic plasticity (RGPs)’ as defined by Mathee *et al*. [39], were detected. Thus, the CLJ1 genome contained 15 regions that are absent from the PA7 genome, and lacked 26 PA7 regions, including some outside recognizable RGPs (Table S4). The differences between PA7 and CLJ1 are presented in Tables S3 and S4. All CLJ1 regions absent from PA7 (CLJ-SR), except CLJ-SR14, were detected in other *P. aeruginosa* strains, some of which are phylogenetically unrelated to the PA7 strain. Strikingly, CLJ-SR14 carries 55 genes, including many that are predicted to encode proteins involved in metabolism and resistance to heavy metals (Table S5), suggesting that the strain is of environmental origin. Moreover, the so-called Dit island previously found in a CF isolate of *P. aeruginosa* [39] was found inserted into the 5′ region of RGP27 at a tRNA^Gly^. The determinants encoded in this island provide the bacteria with the ability to degrade aromatic diterpenes – tricyclic resin acids produced by wounded trees – and to use them as sole carbon and energy source [40]. The Dit island is uncommon in *P. aeruginosa* strains, but is frequently found in a range of soil bacteria such as *Burkholderia xenovorans*, *P. fluorescens* and *Pseudomonas mendocina*. Identification of this island further supports an environmental origin of the CLJ1 strain. Interestingly, most of the genes within RGP7 (pKLC102-like island) were missing from CLJ1 (Table S4).

Four gene clusters have been defined as replacement islands in *P. aeruginosa*; they carry horizontally acquired material and sequences diverge significantly between strains [41, 42]. These gene clusters encode proteins involved in the biosynthesis or post-translational modification of lipopolysaccharide (LPS) O antigen, pyoverdine, pili and flagella. Modifications to three of these clusters can produce the CLJ1-specific phenotype. The CLJ replacement island in RGP60 (harbouring pilin and pilin-modification genes) carries a group I pilin allele. In contrast, PA7 contains a group IV allele [43]. Within RGP9, CLJ1 encodes a b-type flagellin as the principal component of its flagellum, while PA7 has an a-type flagellin [17]. RGP9, containing the flagellin glycosylation genes, and the replacement island in RGP31, bearing the O-specific antigen (OSA) biosynthesis gene cluster, were further modified in CLJ strains (see below).

Among other potential virulence-associated genes, CLJ1 lacks one of the three copies of the type-6 secretion system loci (*PSPA7_2884–2902*) encoding a machine to inject toxins with activity in both prokaryotic and eukaryotic cells [44]. The *plcH* and *plcR* genes, coding for the haemolytic phospholipase C precursor and its accessory protein, respectively, were also absent from the CLJ1 genome. Although the *cupA* fimbrial gene cluster (*CLJ1_2899–2903; PSPA7_3019–3023*) was present in RGP23, the entire *cupE1-6* operon (*PSPA7_5297–5302*), which encodes cell surface fimbriae required to maintain a biofilm structure [45], was missing.

### Evidence of mobile genetic elements in key pathogenic regions

**Fig. 2.**
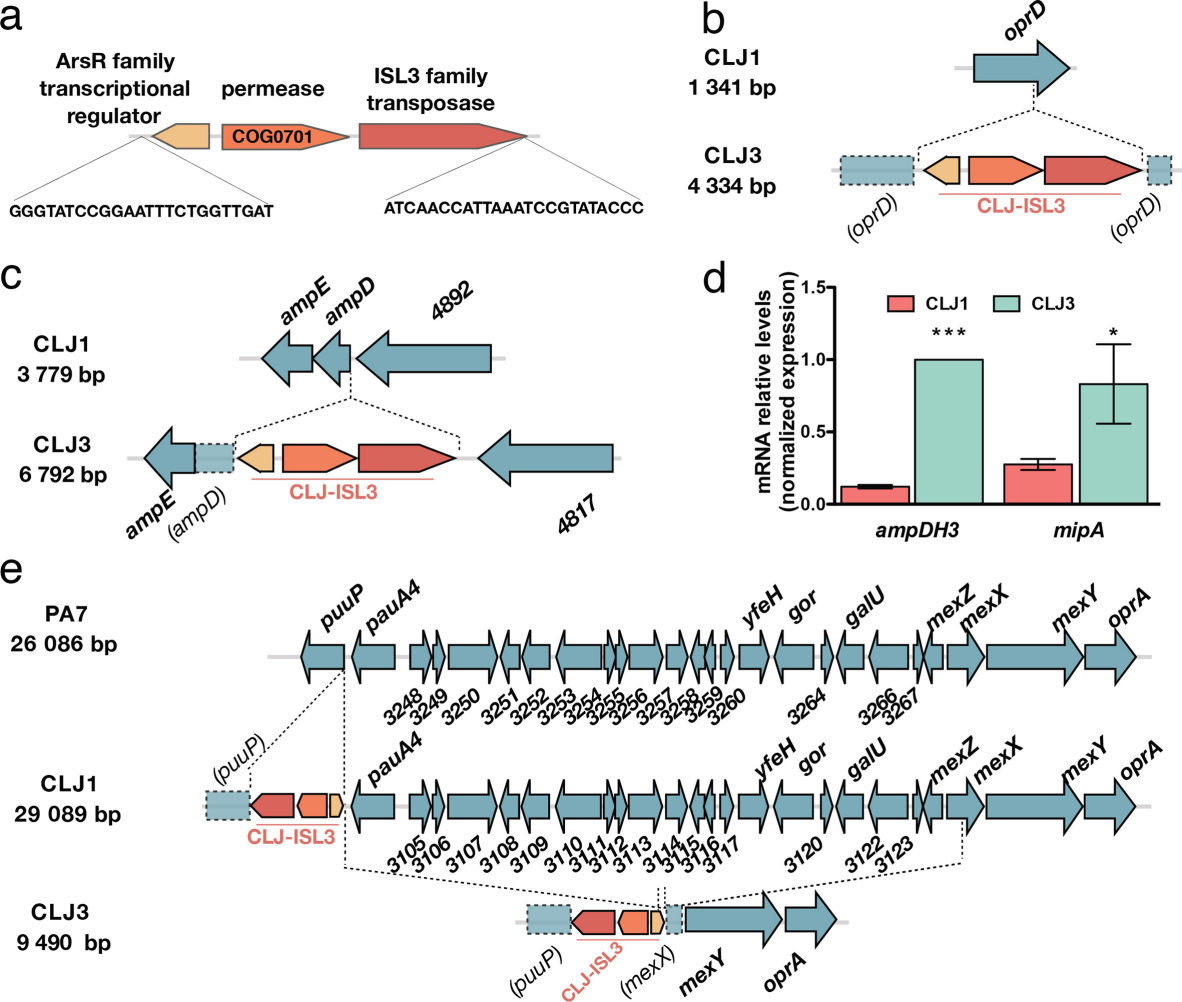
Insertions of CLJ-ISL3 into genes encoding determinants of antibiotic susceptibility. (a) Representation of the 2985 bp CLJ-ISL3, encompassing three genes and with inverted repeats at its extremities. (b) Representation of disruption of *oprD* in CLJ3 by CLJ-ISL3. (c) Location of CLJ-ISL3 in *ampD* in CLJ3. (d) RT-qPCR analysis of the relative expression of *ampDH3* and *mipA* in CLJ1 and CLJ3 strains. Error bars correspond to sem The results of a *t*-test are indicated by asterisks when the difference between the two strains was statistically significant (*P<0.05 or ***P<0.001). (e) Gene organization of the region present in PA7 and CLJ1 but absent from CLJ3. CLJ-ISL3 was found in *puuP* in CLJ1, whereas both *puuP* and *mexX* were disrupted in CLJ3, with the connecting sequence replaced by CLJ-ISL3 (see main text).

The CLJ3 isolate was sampled from the same patient after a series of aggressive antibiotic treatments and displayed different cytotoxicity and antibiotic-sensitivity profiles compared to CLJ1 [16]. The genomes of the two strains were almost identical, with a reciprocal best-hit average nucleotide identity [46] estimated at 99.97 %, and >600 SNPs between them (Fig. 1). When analysing the genomes of CLJ1 and CLJ3, the most striking feature was the presence of multiple copies of a 2985 bp fragment corresponding to a mobile genetic element, absent from the reference PA7 genome. At the nucleotide level, the whole sequence of this element was 99 % identical to ISPst2 from *P. stutzeri*, ISpu12 from *P. putida* and IS1396 from *Serratia marcescens*, found in the ISfinder database [47] (Table S6). Further examination of publicly available genome sequences (NCBI release February 2019) by blastn searching revealed the fragment to be 100 % identical to a part of the pU12A_D plasmid present in the *Escherichia coli* ST131 urinary tract isolate (accession number: PRJNA516746). The complete sequence was also detected in a few other bacterial strains, including a multi-drug-resistant *Acinetobacter baumannii* isolate recovered from bronchoalveolar lavage fluid [48]. The element is an isoform of ISPst2 and belongs to the ISL3 family, and we therefore named it CLJ-ISL3. In addition to coding for a transposase, CLJ-ISL3 encodes a putative seven-transmembrane domain inner-membrane protein (344 aa, 36 kDa) from the permease superfamily cl17795 (with the conserved domain COG0701 [49]) and a putative transcriptional metalloregulator from the ArsR family (Fig. 2a). The element is flanked by a pair of 24 bp imperfect inverted repeat sequences, GGGTATCCGGAATTTCTGGTTGAT (left inverted repeat, IRL) and GGGTATACGGATTTAATGGTTGAT (right inverted repeat, IRR) (Fig. 2a).

Six and 40 copies of CLJ-ISL3 were present in the genomes of CLJ1 and CLJ3, respectively. The six insertions in the CLJ1 genome were revealed by blast mining of the assembled contigs. In the CLJ3 genome, most of the ISs were predicted using a combination of bioinformatics tools, analysis of genome synteny and detection of inverted sequences (Materials and Methods). In agreement with the clonal origin of the strains, all but one of the CLJ-ISL3 in CLJ1 were found in the same location in the CLJ3 genome (Fig. 1, Table S7). MS-based quantitative proteomic analyses revealed a strong increase in expression of the transposase protein in CLJ3 compared to CLJ1, which might be the result of the considerably higher number of ISs within its genome (Table S8). As mobile genetic elements contribute significantly to phenotypic modifications by altering gene expression or by inactivating genes [50, 51], we examined how these insertions affected overall gene expression and specific phenotypes. To do so, we compared CLJ1 and CLJ3 transcriptomes and proteomes using RNA-Seq and MS-based quantitative proteomics, respectively. The proteomes for three different bacterial fractions (whole bacteria, total membranes and secretomes) were analysed. Stringent statistical analyses of extracted data revealed 75 differentially expressed elements between CLJ1 and CLJ3, at both mRNA and protein levels (Fig. 1, Tables S8, S9 and S10). Among these elements, 27 (35 %) were phage-related, while 32 (42 %) were predicted to be localized in bacterial membranes, the periplasm or secreted.

### Contribution of CLJ-ISL3 to antibiotic resistance in the CLJ3 strain

The CLJ1 and CLJ3 strains were recently isolated from a patient treated unsuccessfully with high doses of various antibiotics. CLJ1, isolated before the beginning of the antibiotic therapy, was sensitive to the antibiotics tested, whereas CLJ3 displayed resistance to most of the antibiotics administered to the patient [16]. To gain insights into the mechanisms of antibiotic resistance developed by CLJ3, we examined the genomic data for gene modifications that could explain the switch in phenotypes. Several genes encoding proteins potentially conferring antibiotic resistance were observed to be modified by ISs.

As the patient was given several antibiotics from the β-lactam family (ticarcillin, carbapenem, cephalosporin, etc.) and the CLJ3 isolate developed resistance to all of them, we examined the status of the two major determinants of intrinsic resistance to this group of antibiotics, the outer-membrane porin OprD (CLJ1_4366) and the chromosomally encoded AmpC β-lactamase (CLJ1_0728). The *oprD* gene was found to be interrupted by the CLJ-ISL3 insertion (Fig. 2b), resulting in absence of the protein (Table S8). Loss-of-function mutations or deletions in *oprD* make the cell envelope impermeable to these antibiotics and have been repeatedly reported in isolates from patients undergoing treatment with imipenem or meropenem [52–55]. In addition, CLJ-ISL3 was detected in the 5′ portion of the *ampD* gene (Fig. 2c), encoding the recycling amidase responsible for the production of muropeptide regulators of *ampC* expression [56, 57]. Consequently, although RNA-Seq data indicated no difference in *ampC* expression, CLJ3 proteomes contained significantly greater amounts of the AmpC protein than CLJ1 extracts (Table S8).

Two additional periplasmic proteins involved in peptidoglycan recycling and biosynthesis, the AmpDH3 amidase (CLJ1_5671) [58] and the lytic transglycosylase MltA-interacting protein MipA (CLJ1_3357) [59], were also over-represented in the CLJ3 proteome. In these cases, RT-qPCR data indicated that the corresponding genes were also overexpressed (Fig. 2d, Table S9). Overproduction of these proteins in a clinical strain suggests a role in adaptation of the strain to the host through modulation of peptidoglycan synthesis. However, we were unable to find a link between upregulation of those two genes and CLJ-ISL3 insertion. The molecular mechanisms involved in the increased expression observed and the relevance of their overexpression to bacterial persistence in the host therefore remain to be determined.

All *P. aeruginosa* strains carry genes for multiple efflux pumps. The CLJ1/PA7 clade possesses the locus encoding the MexXY-OprA efflux pump, which can transport multiple antibiotics including fluoroquinolones, aminoglycosides and certain cephalosporins (reviewed by Li and colleagues [60]). However, when compared to CLJ1, an approximately 20 kb deletion in the CLJ3 genome (resulting in loss of 22 genes corresponding to *PSPA7_3247–3268*) was detected, corresponding to elimination of the entire transcriptional repressor *mexZ* gene and truncation of *mexX* (Fig. 2e). The deleted region was replaced by a copy of CLJ-ISL3 that is bordered by truncated *puuP* and *mexX* sequences. One plausible explanation for the genomic arrangement observed in this region in CLJ3 is that the strain was derived from an as-yet unidentified clonal strain that had another copy of CLJ-ISL3 in *mexX*. Recombination between these two IS elements would produce the 20 kb deletion, simultaneously eliminating the repressor and a portion of the *mexX* genes, rendering this efflux pump non-functional. To investigate this hypothesis, we analysed the DR (direct repeat) sequences flanking the CLJ-ISL3 sequences in both CLJ1 and CLJ3 genomes. DRs are short sequences generated during the process of IS insertion, and their analysis can provide evidence of homologous recombination between two IS elements. CLJ-ISL3 inserted in the CLJ1 *puuP* gene was flanked by the 8-bp-long ‘TTCTTTTT’ sequence, corresponding to the classical DR length for ISL3. In agreement with our hypothesis, a hybrid element was found in CLJ3, as the same DR sequence was found to flank one side of the IS (in the *puuP* truncated gene), whereas the sequence 'AATTTTTC' of *mexX* was found on the other side. Truncation of the *puuP* gene, encoding putrescine importer/permease, indicates that the CLJ-ISL3 sequence affects transport of polyamines, which play multiple roles in pathogen biology, including conferring resistance to some antibacterial agents [61].

### Modifications to the OSA cluster due to CLJ-ISL3 insertions

**Fig. 3.**
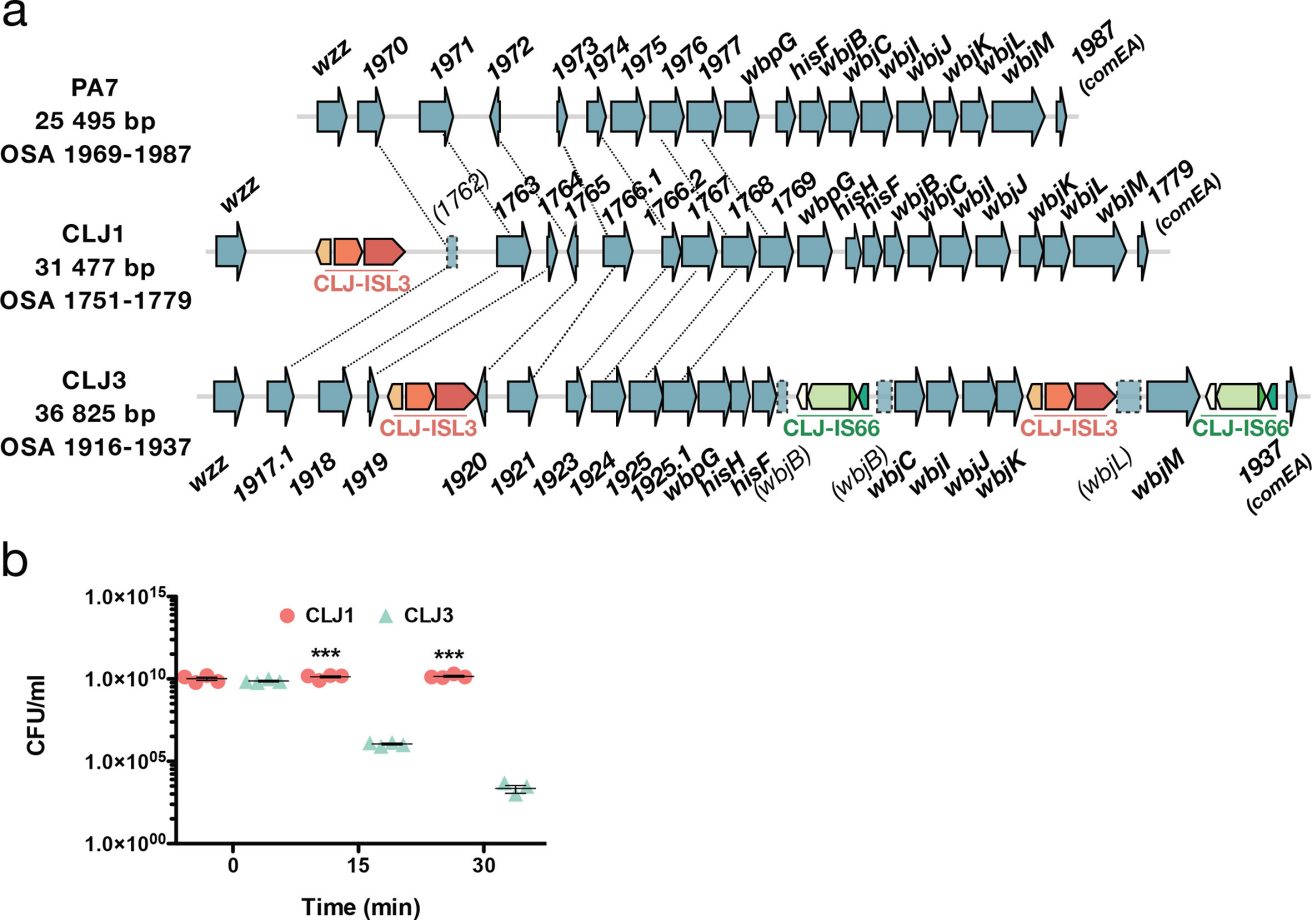
ISs within OSA loci and how they affect serum sensitivity. (a) Comparison of the OSA region in PA7 and CLJ strains. CLJ-ISL3 was found in one location in CLJ1 and at two sites in CLJ3, whereas two copies of CLJ-IS66 were present in CLJ3. Correspondence between non-annotated genes is shown by connecting lines. (b) Kinetics of serum killing of CLJ1 and CLJ3 strains. Bacteria were incubated with human sera for the time indicated, before dilution and spotting on agar plates. Numbers of c.f.u. were counted after incubation at 37 °C for 16 h. Statistical significance was determined using a *t*-test. ***P<0.001.

OSA is a component of LPS and an integral component of the cell envelope of *P. aeruginosa*. The OSA biosynthetic gene cluster in CLJ, RGP31, is similar in content to that of PA7, and both PA7 and CLJ1 belong to serotype O12 [22]. However, insertion of two different ISs affects the gene content and expression levels of the proteins encoded (Fig. 3a). In CLJ1, the CLJ-ISL3 element was detected within the gene encoding NAD-dependent epimerase/dehydratase (CLJ1_1762 corresponding to PSPA7_1970), whereas in CLJ3 two copies were present, one disrupting *wbjL* and the second located in the intergenic region between *CLJ3_1919* and *CLJ3_1920* (*PSPA7_1972*). In addition, in CLJ3, two copies of another IS, sharing 75 % nucleotide identity with ISCARN15 from the IS66 family, were detected within the *wbjB* gene (*CLJ1_1771*; *CLJ3_1930*), and downstream of *wbjM* (*CLJ1_1778*; *CLJ3_1936*). By performing bioinformatic searches for IS extremities in CLJ1 and CLJ3 contigs, this IS, which we named CLJ-IS66, was found to be present in a single location in CLJ1, within the CLJ-SR12 region (*CLJ_4873-CLJ_4876*). An identical insertion was found in CLJ3. CLJ-IS66 within the OSA locus negatively affects expression of the corresponding genes, or stability of the mRNA produced, as higher levels of both mRNA and protein were detected in CLJ1 compared to CLJ3 (Tables S8 and S9).

Interestingly, the deletion at the *mexX-puuP* locus in CLJ3 described above also encompasses the *galU* gene (*CLJ1_3121*, Fig. 2e), which encodes UDP glucose pyrophosphorylase [62]. This enzyme adds sugar moieties onto the inner core on lipid A, serving as an anchor for the full-length LPS. In the absence of *galU*, LPS is truncated and a rough LPS is produced, making bacteria more susceptible to serum-mediated killing and reducing their *in vivo* virulence [63]. The CLJ3 strain was indeed more sensitive to serum than the CLJ1 strain (Fig. 3b). The *in vivo* mechanism leading to selection of a serum-sensitive phenotype is unclear. Modification to LPS structures in *P. aeruginosa* strains chronically infecting CF patients is considered an adaptation mechanism to a more ‘persistent’ lifestyle, by making the LPS molecule less inflammatory [63]. Thus, multiple mechanisms contributed to the final LPS structure in CLJ clones. Those events could have provided strains with numerous advantages during the infection process, such as resistance to antimicrobials or altered recognition by the immune system.

### ISs determine the repertoire of surface appendices

**Fig. 4.**
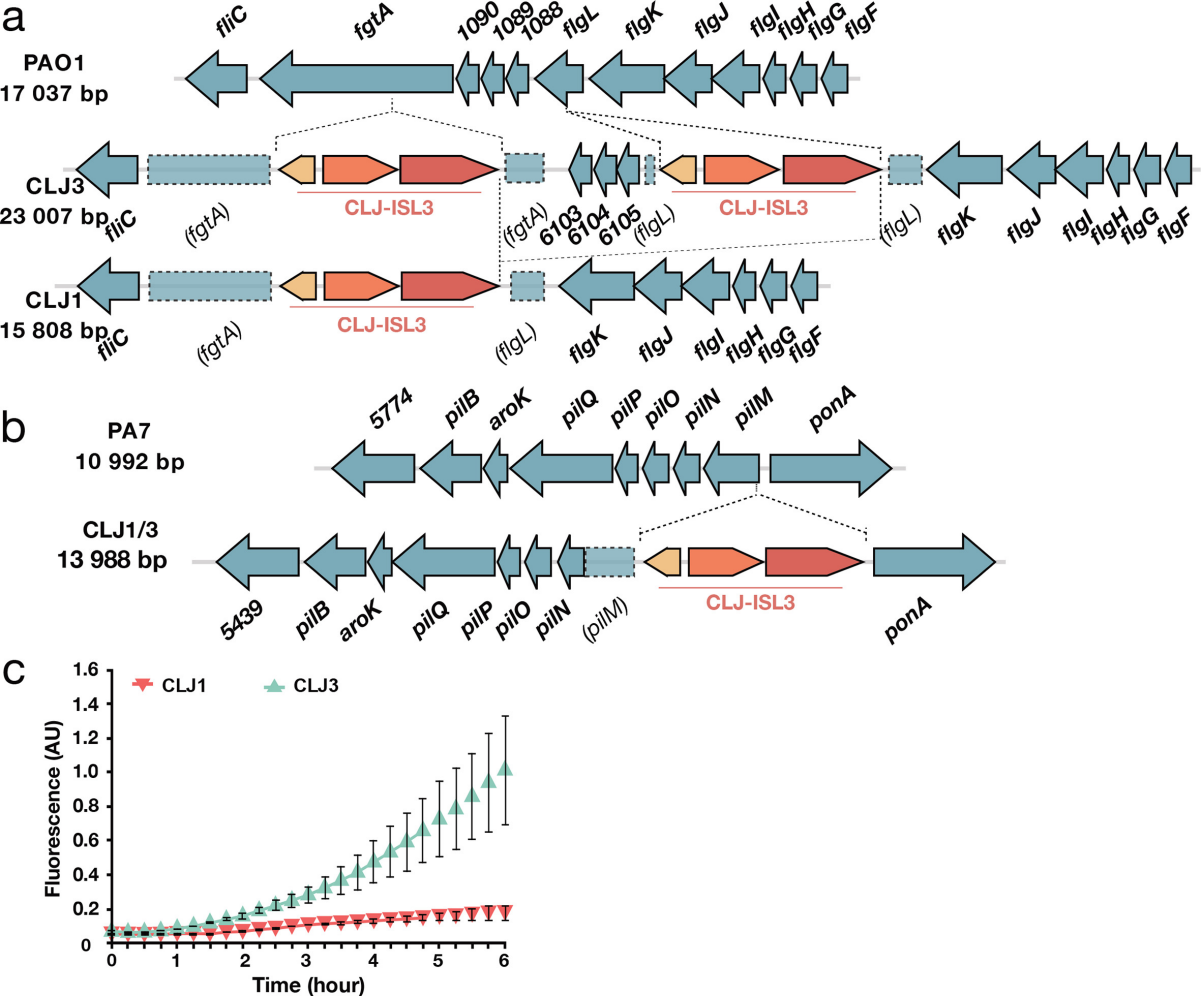
Modifications to components associated with surface appendages. (a) Gene organization of the *flgL* region in PAO1 and the CLJ1 strain. Two CLJ-ISL3, interrupting *fgtA* and *flgL*, were found in the CLJ3 genome that probably recombined in CLJ1, leaving only a single copy of the IS. (b) Representation of the *pilMNOPQ* operon. CLJ-ISL3 was inserted into the *pilM* gene in both CLJ1 and CLJ3. (c) Synthesis of the adhesin CdrA is linked to high intracellular c-di-GMP levels. The c-di-GMP levels in CLJ1 and CLJ3 strains were monitored using the p*cdrA-gfp*(ASV)^c^ plasmid. Fluorescence was measured every 15 min over 6 h of growth. Error bars indicate sd.

Inspection of CLJ1 and CLJ3 genome sequences showed that CLJ-ISL3 has strongly affected the flagellar biosynthetic locus in both strains. The CLJ3 genome revealed an organization and gene content of the flagellar locus similar to that found in PAO1, which synthesizes a b-type flagellum [64], but with two copies of CLJ-ISL3 (Fig. 4a). The first IS interrupts *flgL* (*CLJ1_4222*), encoding the flagellar hook-associated protein through which the flagellum tethers to the cell envelope, whereas the second IS element is in *fgtA* (*CLJ1_4219*), which codes for the flagellin glycosyl transferase. These two ISs seem to have recombined in CLJ1, creating a deletion between the truncated *fgtA* and *flgL* genes (Fig. 4a). In agreement, the DRs flanking the two sides of the CLJ-ISL3 in CLJ1 are different and correspond to that found in CLJ3 in the IS integrated within *fgtA* (‘ATTTTCCA’) and *flgL* (‘CGAAAATA’). This recombination between two ISs suggests that CLJ1 is not the direct ancestor of CLJ3, but that the two strains evolved from a common ancestor. The absence of a flagellum is in agreement with the non-motile phenotype of the strain observed during eukaryotic cell infection and on soft agar [22]. Bacterial flagella are also known to modulate the immune response during the response to infection, through binding of the major flagellar subunit, flagellin, to TLR5, which triggers TLR-dependent signalling and activates expression of pro-inflammatory cytokines [65]. The absence of assembled flagella in the isolates explains why pro-inflammatory cytokine IL-1β and tumour necrosis factor were undetectable in bronchoalveolar lavages from CLJ1-infected mice [23, 25].

As CLJ1 also lacks twitching motility [22], we examined the genomic data for possible mutations in genes encoding type IV pili (T4P) and found an insertion of CLJ-ISL3 within the 5′ part of the *pilM* gene in both the CLJ1 and the CLJ3 genomes (Fig. 4b). This gene encodes a cytoplasmic actin-like protein, and is the first gene in the *pilMNOPQ* operon, which is reported to be essential for both T4P biogenesis and twitching motility [66]. Expression of the entire operon appears to be affected, as no proteins were detected by proteomic analysis, unlike several other Pil proteins encoded by other operons (Table S8). This effect is probably due to the polar effect of the IS element on the downstream genes. This finding was intriguing as the action of Exolysin, responsible for CLJ1 cytotoxicity, relies heavily on T4P in another *exlA* ^+^ strain, IHMA87 [67]. Therefore, we examined CLJ1 proteomes for the presence of other putative adhesive molecules that may substitute for the T4P function during host cell invasion. Based on proteomic datasets, the CLJ1 strain synthesizes components of five two-partner secretion (TPS) systems, the predicted secreted components, some of which are annotated as haemolysins/haemagglutinins, including Exolysin (CLJ1_4479). As expected, Exolysin (ExlA) was found to be more abundant in the secretome produced by CLJ1 than in the secretome from CLJ3 (log_2_FC −1.7, *P=*8.24E-02) (Table S8). CdrA and CdrB (CLJ1_4999 and CLJ1_5000) were significantly overrepresented in the CLJ3 strain according to proteomics and RNA-Seq analyses (Tables S8 and S9). In the PAO1 strain, the adhesin CdrA is regulated by the secondary messenger c-di-GMP, and its expression increases in biofilm-growing conditions [68]. In the strains studied here, we assessed the cellular levels of c-di-GMP using a reporter based on the c-di-GMP-responsive *cdrA* promoter transcriptionally fused to a gene encoding unstable GFP [36]. The p*cdrA-gfp*(ASV)^C^ monitoring plasmid clearly revealed higher levels of the second messenger in CLJ3 during growth (Fig. 4c), suggesting that CLJ3 may have adapted to host conditions by switching to the biofilm lifestyle. Another TpsA protein detected in CLJ secretomes is CLJ1_4560, an HMW-like adhesin named PdtA in the PAO1 strain. PdtA plays a role in *P. aeruginosa* virulence, as demonstrated in the *Caenorhabditis elegans* model of infection [69]. The production of five TPSs, including the protease LepA (CLJ1_4911) [70] and at least one contact-dependent inhibition protein Cdi (CLJ1_2745) [71, 72], in CLJ strains (Table S8) indicates that this family of proteins may play an important role during colonization and infection, but their respective contributions to adhesion, cytotoxicity or inter-bacterial competition during the infectious process will need to be investigated.

### Other putative virulence factors, phages and metabolism

**Fig. 5.**
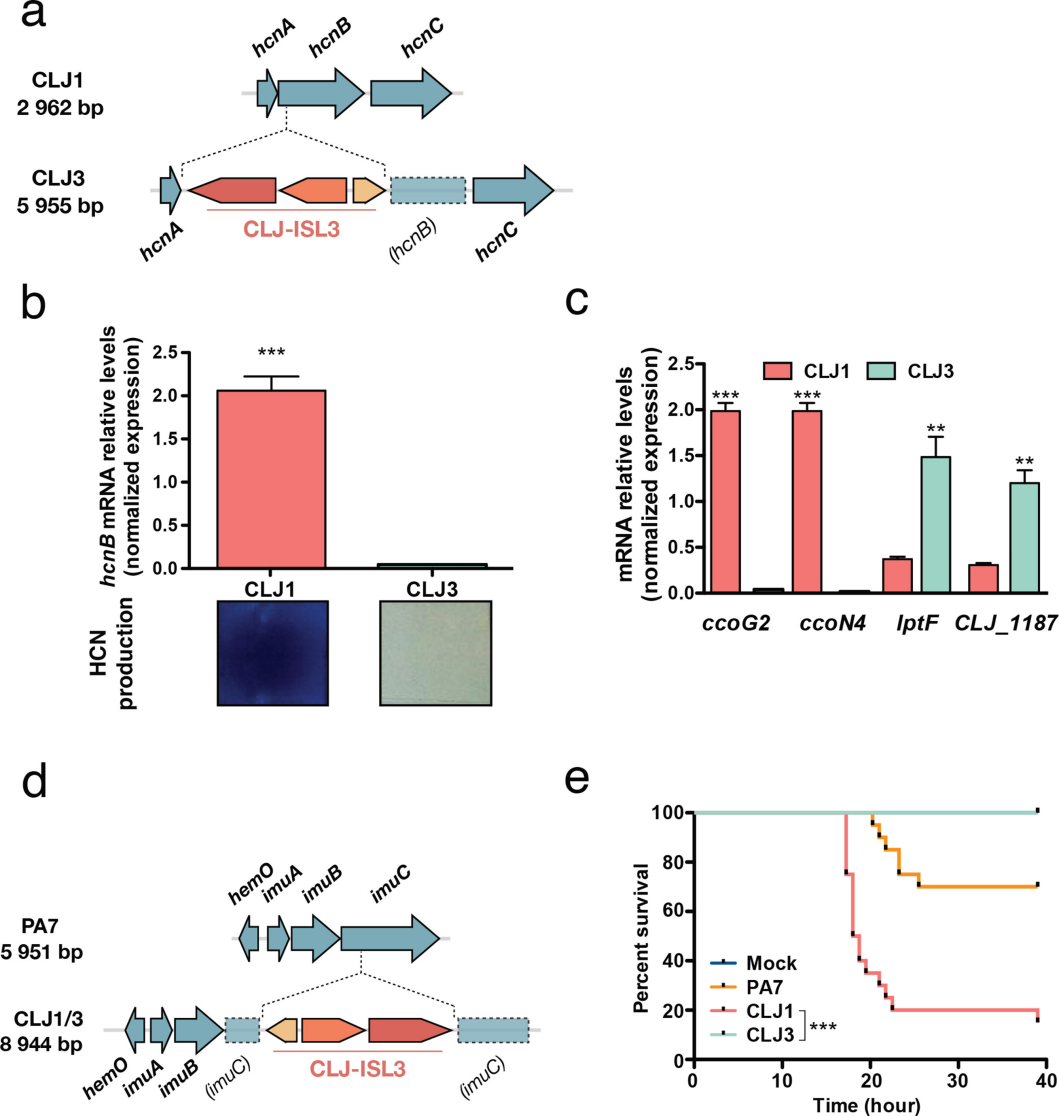
Expression of virulence factors and pathogenicity. (a) The *hcn* operon in CLJ1 and insertion of CLJ-ISL3 into the *hcnB* gene in CLJ3. (b) Relative expression of *hcnB* measured by RT-qPCR and HCN production in the strains indicated. Statistical significance was determined using a *t*-test. ***P<0.001. HCN production was detected by placing a paper impregnated with a reaction mixture containing Cu^2+^ ions above agar plates seeded with bacteria. A white-to-blue colour transition indicates the presence of HCN in the gas phase. (c) RT-qPCR analysis of the relative expression of *ccoG2*, *ccoN4*, *lptF* and CLJ1_*1187* in CLJ1 and CLJ3 strains. Bars, sem. Statistical significance was determined using a *t*-test. **P<0.01 and ***P<0.001. (d) Insertion of CLJ-ISL3 into the *imuABC* operon. (e) Survival of *Galleria* following injection of different strains. Twenty larvae were infected with 5–10 bacteria (estimated from c.f.u. counts) and their survival was monitored over the indicated period. PBS was injected as a vehicle-control. ***P<0.001.

RNA-Seq results, supported by proteomics data, showed increased expression of the enzymes HcnB (CLJ1_2955) and HcnC (CLJ1_2954) in the CLJ1 strain (Table S10). Inspection of the *hcn* operon revealed that the CLJ-ISL3 element was inserted into the 5′ part of the *hcnB* gene in the CLJ3 genome (Fig. 5a), which explains the lack of expression measured by RT-qPCR (Fig. 5b). The *hcn* genes encode the subunits of hydrogen cyanide (HCN) synthase, which produce a toxic secondary metabolite [73]. The HCN produced by the strains was detected using a standard method (see Material and Methods). In agreement with proteomics data, CLJ1 produced HCN in higher quantities than CLJ3 (Fig. 5b, [22]). Many *P. aeruginosa* isolates from individuals with CF produce high levels of HCN [74], and the molecule has even been detected in the sputum of *P. aeruginosa*-infected CF and bronchiectasis patients [75, 76]. HCN also plays a regulatory role, inducing and repressing the expression of other genes [77], including the *PA4129-PA4134* gene cluster in PAO1. This seven-gene cluster (*CLJ1_0701-CLJ1_0702*, *CLJ1_0704-CLJ1_0708*) is expressed at higher levels in CLJ1 compared to CLJ3 (Fig. 1, Tables S8 and S9). These results were confirmed for *ccoG2* and *ccoN4* by RT-qPCR (Fig. 5c). *ccoG2* codes for a cytochrome *c* oxidase accessory protein and *ccoN4* for a cytochrome *c* oxidase subunit, both products being involved in aerobic respiration. The *ccoN4* gene belongs to the *ccoN4Q4* operon, one of the two *ccoNQ* orphan gene clusters present in the *P. aeruginosa* genome. Its upregulation in the cyanogenic CLJ1 is in agreement with a study showing that, although *P. aeruginosa* encodes a cyanide-insensitive oxidase CIO, isoforms of *cbb_3_*-type cytochrome *c* oxidase containing the CcoN4 subunit were produced in response to cyanide, exhibiting higher tolerance towards this poisonous molecule in low-oxygen conditions [78]. Among the other genes in the cluster are found a putative sulfite reductase-encoding gene (*CLJ1_0707*) and a gene (*CLJ1_0708*) coding for an oxidoreductase probably involved in sulfite reduction; however, their exact role is unknown.

Examining the data for differential expression of putative virulence determinants indicated that CLJ3 overproduces Lipotoxin F (LptF, CLJ1_1186), an outer-membrane protein contributing to adhesion to epithelial A549 cells which is known to activate the host inflammatory response [79]. The *lptF* gene is located in a putative operon together with a gene encoding a hypothetical protein predicted to be a lipoprotein located in the periplasm (CLJ1_1187). Proteomics analysis revealed this hypothetical protein to be upregulated in CLJ3 (Table S8). As *lptF* upregulation was not detected by initial RNA-Seq analysis, we performed RT-qPCR assays. Significant overexpression of both *lptF* and *CLJ1_1187*/*CLJ3_1413* was detected in CLJ3 (Fig. 5c), suggesting that Lipotoxin F together with CLJ1_1187 contributed to bacterial adaptation to in-human environments, in line with increased *lptF* expression in CF isolates [80].

Finally, prophages play important roles in *P. aeruginosa* physiology, adaptation and virulence [41, 81, 82]. In the CLJ genomes, at least eight regions related to phages were identified (Table S11), five of which differ or are absent from the PA7 genome (Table S3). Transcriptomic and proteomic approaches revealed 27 phage-related proteins from *CLJ1_0539–0556* in RGP3 and *CLJ1_4296–4314*, including CLJ-SR11, to be significantly more abundant in CLJ3 compared to CLJ1 extracts (Tables S8–S10). Interestingly, both CLJ strains lacked the genes encoding bacteriocins, in particular pyocins S2, S4, and S5. Nevertheless, a homologue of PrtN (CLJ1_0535), an activator of pyocin biosynthetic genes [83], was expressed at higher levels in CLJ3 than in CLJ1. Indeed, the *prtN* gene is located in a region encoding phage-related proteins, all of which are overexpressed in CLJ3 (Fig. 1 and Table S10). The genes for AlpR (CLJ1_4295, CLJ3_4269) and AlpA (CLJ1_4296, CLJ3_4268), transcriptional regulators of a programmed cell death pathway in PAO1 [84], were also identified in both CLJ genomes. Finally, *alpA* and all the genes in the *alpBCDE* lysis cassette were highly expressed in CLJ3 (Fig. 1 and Table S10). The *alp* genes are present in a variable, phage-related region that differs in gene composition from PAO1 and PA7 strains, but is identical to that found in three recently sequenced *exlA* ^+^ isolates: AR441 (GenBank: CP029093.1), AR_0356 (GenBank: CP027169.1) and CR1 (GenBank: CP020560.1).

### CLJ-ISL3 inactivates the *imu* operon encoding translesion synthesis machinery

Another location of the CLJ-ISL3 element in the CLJ1 and CLJ3 genomes was within the *imu* operon, also known as the mutagenesis cassette (Fig. 5d). The *imu* operon encodes the ImuC polymerase (formerly DnaE2) and other components of translesion synthesis (TLS), which can bypass lesions caused by DNA damage. The ImuC polymerase in *Pseudomonas* contributes to tolerance to DNA alkylation agents [85], and inactivation of the operon could limit the accumulation of mutations. Interestingly, *P. aeruginosa* isolates from patients with CF frequently display a hyper-mutator phenotype, primarily linked to inactivation of *mutS*. This phenotype has previously been suggested to be advantageous for bacterial adaptation to the niche in CF lungs [86]. The *mutS* gene was identical and intact in CLJ1 and CLJ3, and the predicted proteins differed from the MutS in PA7 by a single amino acid at position 593 (threonine in PA7, serine in CLJ1/CLJ3). Consequently, the physiological impact of inactivation of the TLS system is unclear. Two additional DNA-repair proteins, RecN (CLJ1_5150) and RecA (CLJ1_1263), were over-represented in CLJ3 at both the transcriptome and the proteome levels (Table S10), suggesting an interplay between different mechanisms to defend against uncontrolled mutational rates induced by a hostile environment. More than 600 SNPs were found between CLJ1 and CLJ3, a rate about six times higher than that recorded for sequential isolates in the same CF patients over 8.8 years in one study [10] and almost ten times higher than the mutations reported in a matched isolate pair, collected from a single patient with a 7.5-year interval in another study [12]. However, yet another study indicated that some strains isolated from non-CF patients could have between 176 and 736 SNPs [9]. Although we could not precisely account for the role of the SNPs detected, they may contribute to differences in gene expression between the two isolates that could not be directly attributed to ISs.

### The two CLJ clones show different pathogenic potential in *G. mellonella*

To assess the overall virulence of the two strains in a whole organism, we used the wax moth (*Galleria*) infection model, and monitored the survival of infected larvae following inoculation with the different strains. Under the same infection conditions, the CLJ1 strain was found to be more virulent than the PA7 strain, and CLJ3 was unable to kill *Galleria* larvae (Fig. 5e). This result indicates that CLJ3, in the process of gaining resistance to antimicrobials, lost its virulence potential, a finding that agrees with our previous observation that CLJ3 is sensitive to serum (Fig. 3b) and less cytotoxic due to loss of its ability to secrete Exolysin [16]. More than 40 CLJ-ISL3 ISs were detected in the CLJ3 genome, some within or upstream of genes encoding hypothetical proteins or putative regulators (Table S7), and some of which may have influenced fitness of the CLJ3 strain in the *Galleria* model of infection. Thus, we found that ISs contributed significantly to the pathogenicity of the CLJ1 strain and to the multi-drug resistance of CLJ3. As the two isolates may have coexisted in the patient’s lungs, we can conclude that ISs determined the overall success of the CLJ lineage in establishing lethal infection.

## Discussion

*P. aeruginosa* strains from the group of taxonomic outliers are abundant in humid environments [18, 19, 87, 88] and, based on a previous study, are considered to be innocuous [89]. Here, we present the results of a multiomics approach applied to two recent clinical isolates, CLJ1 and CLJ3, members of the same group of taxonomic outliers. The results provided insights into genome-wide modifications that provided these bacteria with the tools to successfully colonize and disseminate within the human host. We found the genomes of both strains to be highly dynamic and to evolve within the patient due to high numbers of ISs. General pathogenic and survival traits, e.g. motility, adhesion and resistance to antimicrobials, were modulated by the CLJ-ISL3 element. The later isolate, CLJ3, had acquired resistance toward antibiotics with which the patient was treated during hospitalization, and some ISs directly affected components involved in resistance. Compared to the early colonizer CLJ1, the CLJ3 strain also displayed higher intracellular levels of c-di-GMP, higher expression of the biofilm-associated adhesion protein CdrA, and had lost part of its LPS due to deletion of the *galU* gene. All these features promoted adaptation to a lifestyle associated with chronic infection. Moreover, by bioinformatics screening we identified 36 additional IS elements in the CLJ3 genome, and these probably contributed to other phenotypic changes which were not assessed in this study. In addition to previously known virulence determinants, several of the genes that were differentially expressed here were annotated as ‘hypothetical’ by RAST automated annotation technology. Their contribution to *P. aeruginosa*’s adaptation to life within a human host should now be further explored.

Although the precise origin of the CLJ-ISL3 identified in the CLJ lineage is unknown, we speculate that it was acquired from another bacterial species present in the environment. Indeed, the GC content of the CLJ1-ISL3 sequence is 54.8 %, whereas in the rest of the *P. aeruginosa* genome it is 66.6 %, suggesting recent acquisition by horizontal transfer. Initial disruption of the genes encoding flagellar components and pili by this sequence may have allowed the CLJ strain to evade the patient’s immune defences. By increasing its capacity to secrete the pore-forming toxin Exolysin, the strain acquired an additional advantage allowing it to further damage the host epithelium and endothelial tissues to promote its dissemination.

Previous studies have indicated that the contribution of ISs to the adaptation of *P. aeruginosa* to the environment in patients with CF is low, with limited numbers of transposition events occurring during chronic infection [90]. This finding contrasts with reports on *in vitro* evolution experiments with *E. coli* [91, 92]. A search of the *Pseudomonas* database [28] with the nucleotide sequence of the CLJ1-ISL3 fragment revealed a total of 12 strains bearing 100 % identical fragments. Eight of these strains were isolated at Copenhagen University Hospital [93], and the four others in a Seattle-based hospital intensive care units [94]. The precise positions of CLJ-ISL3 in those genomes and the phenotypes produced by the disrupted gene(s) are currently unknown, but this knowledge could provide insights into whether and how the CLJ-ISL3 sequence contributes to the colonization process and the adaptation of those strains to a particular infectious niche. More recent genomic characterization of environmental *P. aeruginosa* isolates from dental unit waterlines showed that, in addition to the OSA loci, IS*Pa*11 altered the genes for two master regulators, LasR and GacS, supporting the idea of a potential for ecological adaptation of *P. aeruginosa* through the integration of mobile elements [95]. Thus, in addition to small nucleotide changes in pathoadaptive genes, mobile genetic elements could drive the emergence of phenotypic traits allowing *P. aeruginosa* to adapt to the niche they encounter, and undoubtedly contribute to strain-specific pathogenicity. The overall contribution of ISs to the increase in the bacterial pathogenic arsenal is quite likely to be underestimated as we currently have limited access to closed bacterial genomes. New DNA sequencing technologies accessing genome fragments measuring tens or even hundreds of kilobases should shed light on the overall impact of mobile elements throughout bacterial evolution.

## Supplementary Data

Supplementary material 1

Supplementary material 2
