# Supplementary material for: Insertion sequences drive the emergence of a highly adapted human pathogen
Source: Microb Genom. 2019 Apr 4;6(9):mgen000265. doi: 10.1099/mgen.0.000265 (PMC7643977; doi:10.1099/mgen.0.000265)
Supplement: Supplementary material 1 [file mgen-6-265-s001.pdf]

Supporting Information

Insertion sequences drive the emergence of a highly adapted human pathogen

Erwin Sentausa[1#], Pauline Basso[1+], Alice Berry[1], Annie Adrait[2], Gwendoline Bellement[1,2∞], Yohann Couté[2], Stephen Lory[3], Sylvie Elsen[1]* and Ina Attrée[1]*

*Corresponding authors: ina.attree-delic@cea.fr; sylvie.elsen@cea.fr.

The supplementary information includes:

Supplementary Materials and Methods

Supplementary References

Supplementary Figure S1

Supplementary Tables S1 to S11

Supplementary Materials and Methods

Genome sequencing, assembly, annotation and comparison

Bacterial DNA was extracted using the EZ-10 Spin Column Bacterial Genomic DNA Miniprep Kit (Bio Basic Inc., Canada). DNA extracted from CLJ1 bacteria was sequenced on Illumina HiSeq 2000 systems at the Beijing Genomics Institute (China) in 2×50 base-pairs (bp) paired-end mode, generating 12,333,368 reads with a genome coverage of 90-100x. Reads were trimmed to remove low quality sequences (limit = 0.05) and ambiguous nucleotides (maximum two nucleotides allowed). Reads were then assembled *de novo* using CLC Genomics Workbench 9.0 (Qiagen, Aarhus, Denmark). In parallel, PacBio (Base Clear, Leiden, Netherlands) technology was also used, providing 114,707 reads. The resulting contigs were then combined with the Illumina reads using the “Join Contigs” function in the CLC Genome Finishing Module version 1.6.1 (Qiagen). Contigs or scaffolds consisting of fewer than 100 reads were filtered out, and BLAST [1] searches were performed to identify and remove those that showed no match to *Pseudomonas* sequences in GenBank [2]. CLJ3 genomic DNA was sequenced using Illumina MiSeq at the Biopolymers Facility, Harvard Medical School, Boston, USA, in 150-bp single-end runs, generating 2,495,173 reads. Quality trimming was performed using CLC Genomics Workbench 9.0, applying the same parameters as those used for CLJ1, followed by *de novo* assembly with a minimal contig length of 1 kb. The order of both CLJ1 and CLJ3 scaffolds or contigs was determined based on the genome sequence of PA7 strain using the “move contigs” tool in Mauve Genome Alignment Software (version snapshot_2015-02-13) [3]. Average nucleotide identity between genomes was estimated using the ANI calculator tool on the enveomics collection server [4] with minimum alignment length,

identity, and number set to 700 bp, 70%, and 50, respectively, using a 1,000-bp window size and 200-bp step size. SNPs between CLJ1 and CLJ3 genomes were detected by mapping trimmed CLJ3 reads to the CLJ1 genome in CLC Genomics Workbench 9.0, and then using the Basic Variant Detection 1.71 tool in the Workbench with the following parameters: Ploidy = 1, Ignore positions with coverage above = 100,000, Restrict calling to target regions = Not set, Ignore broken pairs = Yes, Ignore non-specific matches = Reads, Minimum coverage = 10, Minimum count = 2, Minimum frequency (%) = 35.0, Base quality filter = Yes, Neighborhood radius = 5, Minimum central quality = 20, Minimum neighborhood quality = 15, Read direction filter = No, Relative read direction filter = Yes, Significance (%) = 1.0, Read position filter = No, Remove pyro-error variants = No. Clusters of Orthologous Groups (COG) annotation was performed using the WebMGA server [5] with an E-value cutoff of 0.001. Subcellular localizations of PA7 proteins were retrieved from the Pseudomonas Genome Database, whereas those of CLJ1 and CLJ3 proteins were predicted using PSORTb version 3.0.2 [6] and LocTree3 [7]. Orthologous genes across the strains were identified using OrthoMCL version 2.0.9 [8], with a BLASTp E-value cutoff of 1×10^{-5} and the default Markov cluster algorithm (MCL) inflation parameter of 1.5.

Transcriptome Analysis

The Illumina libraries were prepared and sequenced at the Biopolymer Facility, Harvard Medical School, Boston, USA. Illumina HiSeq 2500 was used for the sequencing with 50-bp single-end runs, generating 3,225,727 and 21,633,018 reads for CLJ1 and 2,139,035 and 56,804,547 reads for CLJ3. For each replicate, raw RNA-Seq reads were trimmed in CLC Genomics Workbench 9.0 using the same parameters as for genome sequencing

reads. The trimmed reads were then mapped to the annotated CLJ1 genome using the RNA-Seq analysis tool. The total number of reads mapped to the genes was incorporated into a table and analyzed as raw counts using the DESeq2 differential expression analysis pipeline (87). DESeq2 performs internal normalization for sequencing depth and RNA composition using the median of ratios method. DESeq2 then fits negative binomial generalized linear models for each gene and uses the Wald test for significance testing. Genes that were differentially expressed between CLJ1 and CLJ3 were identified after applying a 5% False Discovery Rate (FDR) filter.

Proteomics

Sample preparation. Whole-cell extracts. Overnight cultures of CLJ1 were diluted to an OD_{600} of 0.1 in 30 mL culture broth and then the cultures were incubated at 37 °C under shaking until they reached OD_{600} 0.8. CLJ3 was left overnight at room temperature and then cultured at 37 °C under shaking to OD_{600} 0.8. The 30-mL bacterial cultures were then centrifuged at 6,000 rpm, 4 °C for 10 min, and supernatants were filtered on 0.22-µm filters. Total membranes separation. Pellets were re-suspended in 1 mL of 10 mM Tris-HCl (pH8), 20% sucrose buffer supplemented with protease inhibitors cocktail (PIC, Roche, Basel, Switzerland) and cells were disrupted by sonication. Intact bacteria were removed by centrifugation at 8,000 rpm for 10 min at 4 °C. The total membrane fraction was then collected by ultracentrifugation at 200,000 g for 1 h at 4 °C. The pelleted fraction was washed twice with 1 mL of 10 mM Tris-HCl, 20 mM $MgCl_2$, pH8, supplemented with PIC, and re-suspended in 500 µL of the same buffer. Supernatant fraction. Proteins contained in supernatants were precipitated by a TCA-sarkosyl method (0.5% final

volume of sarkosyl and 7.5% final volume of TCA) for 2 h on ice and centrifuged at 12,000 rpm for 15 min at 4°C. Pellets were washed twice with tetrahydrofuran, and re-suspended in 50 µL of Laemmli loading buffer. Samples prepared in triplicate were then analyzed by SDS-PAGE and immunoblotting using antibodies directed against three synthetic ExlA peptides ([9], 1:1 000); against RpoA (1:5 000), as a control for whole-cell; against TagQ ([10], 1:10 000), to track the membrane fraction; and against DsbA (1:2 000), to monitor the periplasm fraction. Antibody binding was revealed using appropriate HRP-coupled secondary antibodies, anti-rabbit (1:50 000) and anti-mouse (1:5 000) (Sigma-Aldrich). Western blots were developed using Luminata Crescendo Western HRP (Millipore) substrate.

Mass spectrometry-based quantitative proteomic analyses. Protein extracts were prepared as described in [11]. Briefly, proteins were stacked in the top of an SDS-PAGE gel (NuPAGE 4-12%, ThermoFisher Scientific), and stained with Coomassie blue (R250, Bio-Rad) before in-gel digestion using modified trypsin (Promega, sequencing grade). Resulting peptides were analyzed by nanoliquid chromatography coupled to tandem mass spectrometry (Ultimate 3000 coupled to LTQ-Orbitrap Velos Pro, Thermo Scientific) using a 120-min gradient (2 analytical replicates per biological replicate). RAW files were processed using MaxQuant [12] version 1.5.3.30. The protein contents of total, membrane, and secretome proteomes from CLJ1 and CLJ3 were analyzed independently. Spectra were searched against an in-house CLJ database and the frequently-observed contaminants database included in MaxQuant. Trypsin was selected as the enzyme, and two missed cleavages were allowed. Peptide modifications allowed during the search were: carbamidomethylation (C, fixed), acetyl (Protein N-ter, variable)

and oxidation (M, variable). Minimum peptide length was set to seven amino acids. Minimum number of peptides, razor + unique peptides and unique peptides were all set to 1. Maximum FDRs - calculated by employing a reverse database strategy - were set to 0.01 at peptide and protein levels. Intensity-based absolute quantification values iBAQ [13] were calculated from MS intensities of unique+razor peptides. Statistical analyses were performed using ProStaR [14]. Proteins identified in the reverse and contaminant databases, proteins only identified by site (corresponding to proteins only identified by peptides that carry one or more modified amino acids), and proteins exhibiting fewer than three iBAQ values in any one condition were discarded from the list. After log2 transformation, intensity values were normalized by median centering before imputing missing values (missing values were replaced by the 2.5 percentile value for each column); statistical testing was conducted using a *limma* t-test. Differentially-recovered proteins were sorted out using a log2(fold change) cutoff of 2 and an FDR threshold on remaining p-values of 1%, applying the Benjamini-Hochberg procedure. The various lists were then combined. In total, this procedure produced a list of 2,852 quantified proteins.

RT-qPCR

Yield, purity and integrity of RNA were evaluated on a Nanodrop device and by agarose gel migration. Complementary DNA was synthesized from 3 µg of RNA using the SuperScript III First-Strand Synthesis System (Invitrogen), with or without SuperScript III RT enzyme to check for the presence of genomic DNA. The CFX96 Real-Time system (Bio-Rad) was used to amplify the cDNA, and quantification was performed using SYBR green fluorescent molecules. cDNA was incubated with 5 µL of Gotaq qPCR master mix (Promega), and specific reverse and forward primers were used at a final concentration

of 125 nM in a total volume of 10 µL. Cycle parameters for the real-time PCR were: 95 °C for 2 min, 40 cycles of 95 °C for 15 s and 60 °C for 45 s, and finally a melting curve from 65 °C to 95 °C by 0.5-°C increments for 5 s to assess the specificity of the amplification. To generate standard curves, serial dilutions of pooled cDNA from both CLJ strains were used. Experiments were performed with three biological samples for each strain, in duplicate, and the results were analyzed with the CFX manager software (Bio-Rad). The relative expression of mRNAs was calculated using the ΔΔCq method after normalizing to *rpoD* reference Cq values.

Supplementary References

1. **Altschul SF, Madden TL, Schaffer AA, Zhang J, Zhang Z et al.** Gapped BLAST and PSI-BLAST: a new generation of protein database search programs. *Nucleic acids research* 1997;25(17):3389-3402.
2. **Clark K, Karsch-Mizrachi I, Lipman DJ, Ostell J, Sayers EW**. GenBank. *Nucleic acids research* 2016;44(D1):D67-72.
3. **Darling AC, Mau B, Blattner FR, Perna NT**. Mauve: multiple alignment of conserved genomic sequence with rearrangements. *Genome research* 2004;14(7):1394-1403.
4. **Rodriguez-R LM, Konstantinidis KT**. The enveomics collection: a toolbox for specialized analyses of microbial genomes and metagenomes. *PeerJ Preprints* 2016;4:e1900v1.
5. **Wu S, Zhu Z, Fu L, Niu B, Li W**. WebMGA: a customizable web server for fast metagenomic sequence analysis. *BMC genomics* 2011;12:444.
6. **Yu NY, Wagner JR, Laird MR, Melli G, Rey S et al.** PSORTb 3.0: improved protein subcellular localization prediction with refined localization subcategories and predictive capabilities for all prokaryotes. *Bioinformatics* 2010;26(13):1608-1615.
7. **Goldberg T, Hecht M, Hamp T, Karl T, Yachdav G et al.** LocTree3 prediction of localization. *Nucleic acids research* 2014;42(Web Server issue):W350-355.

8. **Li L, Stoeckert CJ, Jr., Roos DS**. OrthoMCL: identification of ortholog groups for eukaryotic genomes. *Genome research* 2003;13(9):2178-2189.
9. **Elsen S, Huber P, Bouillot S, Coute Y, Fournier P et al.** A type III secretion negative clinical strain of Pseudomonas aeruginosa employs a two-partner secreted exolysin to induce hemorrhagic pneumonia. *Cell host & microbe* 2014;15(2):164-176.
10. **Casabona MG, Robert-Genthon M, Grunwald D, Attree I**. Defining Lipoprotein Localisation by Fluorescence Microscopy. *Methods in molecular biology* 2017;1615:65-74.
11. **Casabona MG, Vandenbrouck Y, Attree I, Coute Y**. Proteomic characterization of Pseudomonas aeruginosa PAO1 inner membrane. *Proteomics* 2013;13(16):2419-2423.
12. **Cox J, Mann M**. MaxQuant enables high peptide identification rates, individualized p.p.b.-range mass accuracies and proteome-wide protein quantification. *Nature biotechnology* 2008;26(12):1367-1372.
13. **Schwanhausser B, Busse D, Li N, Dittmar G, Schuchhardt J et al.** Global quantification of mammalian gene expression control. *Nature* 2011;473(7347):337-342.
14. **Wieczorek S, Combes F, Lazar C, Giai Gianetto Q, Gatto L et al.** DAPAR & ProStaR: software to perform statistical analyses in quantitative discovery proteomics. *Bioinformatics* 2017;33(1):135-136.

Figure S1 Legend

Fig. S1 Details of whole-genome comparison between PA7 and CLJ strains

The outer ring shows all the genes in the strains colored according to their COG (Clusters of Orthologous Groups) functional categories, as listed in the legend. The three other rings represent the PA7 (orange), CLJ3 (green), and CLJ1 (red) genomes. The color code was as follows: white, the gene was absent from the genome; gray, the gene or its homolog was present in a different position in the genome; black in the CLJ rings

correspond to gaps between contigs. The inner labels indicate CLJ-specific regions, whereas the outer labels are PA7-specific regions (represented in RGPs or PSPA7 locus numbers) described in Table S3 and Table S4, respectively.

Table S1 Genome assembly and annotation statistics

Table S2 RT-qPCR primers used

Table S3 Specific regions of CLJ compared to PA7

Table S4 Specific regions of PA7 compared to CLJ

Table S5 Genes in CLJ-SR14

Table S6 BLASTN search result of CLJ-ISL3 (2985 bp) against the ISfinder database with E-value of 0

Table S7 Prediction of CLJ-ISL3 insertions between contigs of CLJ3, as described in Materials and Methods

Table S8 Differential proteomic analysis between CLJ1 and CLJ3 in total proteome, membrane proteome, and secretome proteome

Table S9 Differential gene expression analysis between CLJ1 and CLJ3

Table S10 Genes/proteins that are statistically significantly differentially expressed between CLJ1 and CLJ3 in both RNA-Seq and at least one of the proteomics datasets

Table S11 CLJ phage-related regions

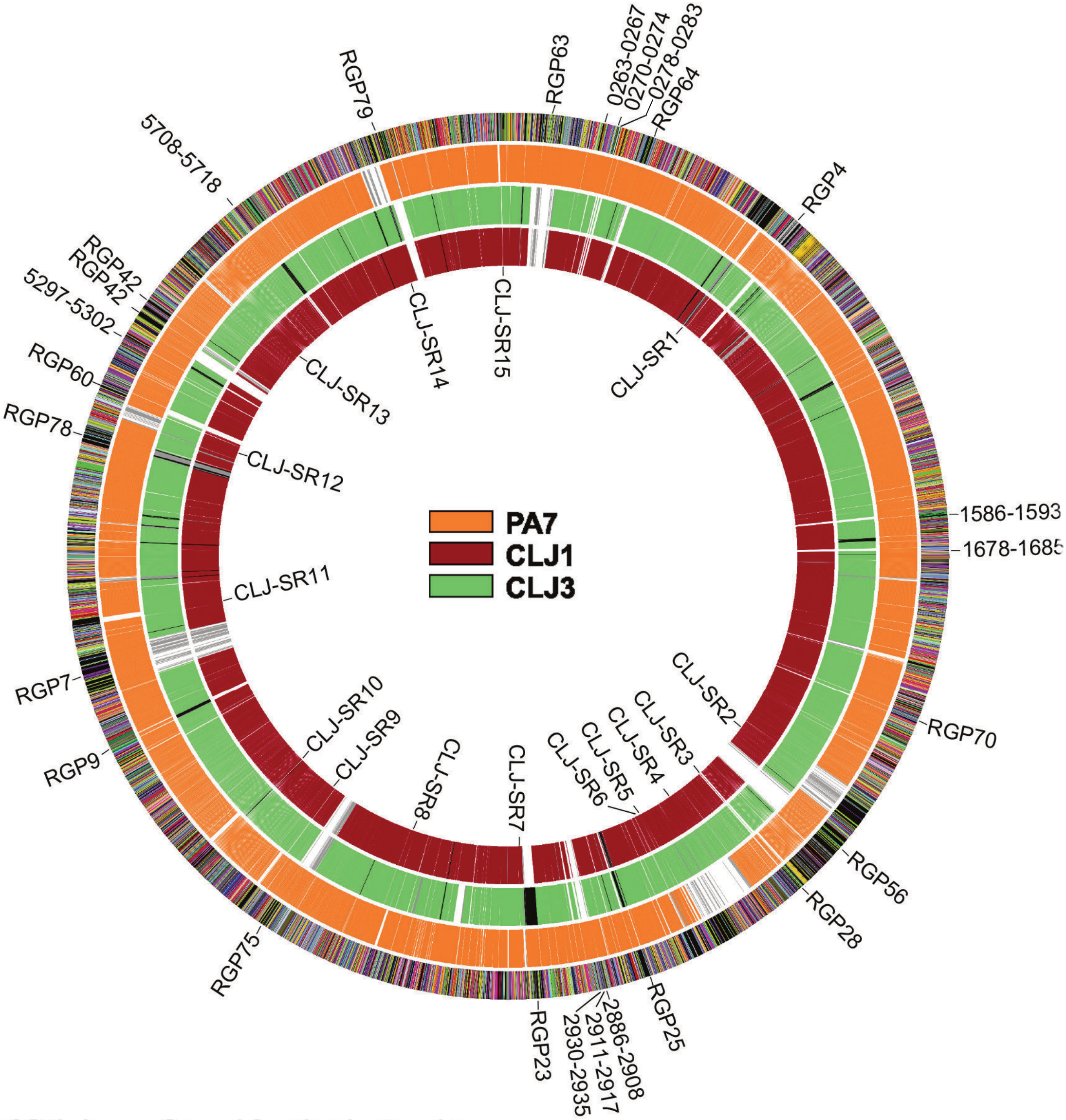

COG (Clusters of Orthologous Groups) functional categories

- RNA processing and modification
- Energy production and conversion
- Cell cycle control, cell division, chromosome partitioning
- Amino acid transport and metabolism
- Nucleotide transport and metabolism
- Carbohydrate transport and metabolism
- Coenzyme transport and metabolism
- Lipid transport and metabolism
- Translation, ribosomal structure and biogenesis
- Transcription
- Replication, recombination and repair
- Cell wall/membrane/envelope biogenesis
- Cell motility
- Posttranslational modification, protein turnover, chaperones
- Inorganic ion transport and metabolism
- Secondary metabolites biosynthesis, transport and catabolism
- Signal transduction mechanisms
- Intracellular trafficking, secretion, and vesicular transport
- Defense mechanisms
- Multiple classes
- General function prediction only
- Function unknown
- No COG

Figure S1

Table S1. Genome assembly and annotation statistics

	CLJ1	CLJ3
**Number of contigs/scaffolds**	68	135
**Average coverage (×)**	98.3	96.9
**Contig/scaffold size (bp)**	201-847201	1025-297138
**Average contig/scaffold size (bp)**	95801	47065
**Contig/scaffold N50 (bp)**	456227	97517
**Total genome size (bp)**	6514448	6353726
**G+C content (%)**	66.6	66.6
**Protein coding sequences**	6259	6107
**tRNA**	62	57
**rRNA**	9	3

Table S2. RT-qPCR primers used

Name	5'-3' sequence	Amplicon size
qPCR-hcnB-F<br>qPCR-hcnB-R	TACGGTGATCTGCCGTTGTG<br>GATCGCTGCAATAGCCGATG	152 bp
qPCR-pvdE-F<br>qPCR-pvdE-R	CCAATCCCGAACCCTACCTG<br>TTCTCGCCGACGATGAAGAG	184 bp
qPCR-ampDH3-F<br>qPCR-ampDH3-R	GCGACAACCTCAACGACACC<br>CATTCTTCGGCGTCATGTCC	165 bp
qPCR-2958-F2<br>qPCR-2958-R2	CGGCATCGAGCACTGCTACT<br>CCAGTTCGTGGCCGATGAT	152 bp
qPCR-ccoG2-F<br>qPCR-ccoG2-R	GCTGGACCTGGAAAGCCTGT<br>GGCGTCGTAGGAAACGATCA	160 bp
qPCR-ccoN4-F<br>qPCR-ccoN4-R	TGGGCAATACCACCACCAAG<br>CGGTGGTCAGGATGAACGAG	171 bp
oprD-up<br>oprD-up	GGGTTCATCGAAGACAGCAG<br>TGCCTTGGGTGAAGCCGGATT	139 bp
uvrD-up<br>uvrD-down	CATATCCTGGTGGACGAGTTCC<br>CGCTGAACTGCTGGATGTTCTC	160 bp
rpoD-qPCR F1<br>rpoD-qPCR R1	GCG-CAA-CAG-CAA-TCT-CGT-CT<br>ATC-CGG-GGC-TGT-CTC-GAA-TA	177 bp

Table S3. Specific regions of CLJ compared to PA7

Region	RGP[1]	CLJ1 locus	Number of genes	Features
**CLJ-SR1**	RGP66	0487-0494	8	Phage-related
**CLJ-SR2**	RGP29*	2128-2214	87	PAGI-2-like island
**CLJ-SR3**	RGP28	2316-2322	6	Mobile element proteins
**CLJ-SR4**	RGP27	2415-2573	160	Dit island
**CLJ-SR5**	RGP72	2579-2583	5	D-galactonate catabolism
**CLJ-SR6**	RGP26	2615-2621	7	Phage-related; lytic enzymes
**CLJ-SR7**	RGP23	2917-2924	7	Cup fimbria, Mobile element proteins; possible DNA helicase
**CLJ-SR8**		3303-3309	7	ABC transporter ATP-binding protein
**CLJ-SR9**		3607-3616	9	
**CLJ-SR10**	RGP15	3774-3784	11	Threonine dehydratase; ABC transporter proteins
**CLJ-SR11**		4314-4301	14	AlpBCDE-lysis cassette, phage-related
**CLJ-SR12**		4838-4876	39	Pyrroloquinoline quinone synthesis proteins
**CLJ-SR13**		5192-5203	10	Phage related; accessory cholera enterotoxin
**CLJ-SR14**		5811-5871	55	Heavy metal resistance
**CLJ-SR15**		6156-6161	6	Phage-related

[1]RGP: region of genomic plasticity, as defined in Mathee *et al*. (2008). The numbering corresponds to published RGPs. *CLJ strains do not share any genes with PA7 in the RGP.

Table S4. Specific regions of PA7 compared to CLJ

PSPA7 numbering	Number of genes	RGP[1]	Features
**0069-0139**	71	RGP63*	Type I restriction-modification system; mercury resistance cluster
**0263-0267**	5		Sulfate ester transport system
**0270-0274**	5		
**0278-0283**	6		*tonB2-exbB1-exbD1* operon
**0355-0369**	15	RGP64*	Phage-related
**0776-0788**	12	RGP4*	Phage-related
**1586-1593**	8		Polymyxin and cationic antimicrobial peptide resistance cluster
**1678-1685**	8		Sulfur starvation utilization operon
**2109-2112**	4	RGP70	Probable transposase
**2363-2435**	73	RGP56*	Phage-related
**2515-2526**	10	RGP28	
**2790-2795**	6	RGP25	Hemagglutinins
**2886-2908**	23		Hcp secretion island-3 encoded type VI secretion system (H3-T6SS)
**2911-2917**	7		Methionine ABC transporters; monooxygenases
**2930-2935**	6		Alkanesulfonate assimilation; nitrate and nitrite ammonification
**3034-3071**	37	RGP23	Pyocin killing protein; cup fimbria, phage-related
**3695-3747**	53	RGP75*	Conjugal transfer protein cluster; resistance genes; transcriptional regulators
**4281-4289**	9	RGP9	Rieske family iron-sulfur cluster-binding protein
**4427-4530**	103	RGP7	pKLC102-like, Type IV B pilus protein cluster
**5053-5075**	23	RGP78	Phage-related
**5143-5160**	17	RGP60*	Phage-related, type IV pilin accessory protein
**5297-5302**	6		Fimbrial chaperone/usher pathway E operon

**5324-5357**	34	RGP42	Mobile element proteins; Streptomycin phosphotransferase
**5364-5378**	15	RGP42	Phage-related
**5708-5718**	11		Arginine:pyruvate transaminase; 2-ketoarginine decarboxylase
**6033-6063**	31	RGP79*	Type I restriction-modification system

[1]RGP: region of genomic plasticity, as defined in Mathee *et al*. (2008). The numbering corresponds to published RGPs. *PA7 does not share any genes with CLJ strains in the RGP.

Table S5. Genes in CLJ-SR14

PA7	CLJ1	CLJ3	RAST annotation
NA	CLJ1_5811	CLJ3_5608	MG(2+) CHELATASE FAMILY PROTEIN / ComM-related protein
NA	CLJ1_5814	CLJ3_5609	hypothetical protein
NA	CLJ1_5815	CLJ3_5610	hypothetical protein
NA	CLJ1_5817	CLJ3_0003	hypothetical protein
NA	CLJ1_5818	CLJ3_0004	Error-prone, lesion bypass DNA polymerase V (UmuC)
ns	CLJ1_5819	CLJ3_0005	hypothetical protein
ns	CLJ1_5820	CLJ3_0006	Mercuric ion reductase (EC 1.16.1.1)
ns	CLJ1_5821	CLJ3_0007	Mercuric transport protein, MerC
ns	CLJ1_5822	CLJ3_0008	Periplasmic mercury(+2) binding protein
ns	CLJ1_5823	CLJ3_0009	Mercuric transport protein, MerT
ns	CLJ1_5824	CLJ3_0010	Mercuric resistance operon regulatory protein
ns	CLJ1_5825	CLJ3_0011	hypothetical protein
NA	CLJ1_5826	CLJ3_0012	Sterol desaturase
NA	CLJ1_5827	CLJ3_0013	Transcriptional regulator, AraC family
NA	CLJ1_5828	CLJ3_0014	Lipoprotein signal peptidase (EC 3.4.23.36)
NA	CLJ1_5829	CLJ3_0015	hypothetical protein
NA	CLJ1_5830	CLJ3_0016	Cobalt-zinc-cadmium resistance protein CzcD
NA	CLJ1_5831	CLJ3_0017	COG3267: Type II secretory pathway, component ExeA (predicted ATPase)
NA	CLJ1_5832	CLJ3_0018	FIG131328: Predicted ATP-dependent endonuclease of the OLD family
ns	CLJ1_5833	CLJ3_0019	Mobile element protein
ns	CLJ1_5834	CLJ3_0020	Mobile element protein
NA	CLJ1_5836	CLJ3_6032	transcriptional regulator MvaT, P16 subunit, putative
ns	CLJ1_5837	CLJ3_6031	Gifsy-2 prophage protein
ns	CLJ1_5838	CLJ3_6030	Error-prone repair protein UmuD
ns	CLJ1_5839	CLJ3_6029	Error-prone, lesion bypass DNA polymerase V (UmuC)
NA	CLJ1_5840	CLJ3_6028	putative (L31491) ORF2; putative [Plasmid pTOM9]
NA	CLJ1_5841	CLJ3_6027	putative ORF1 [Plasmid pTOM9]
NA	CLJ1_5842	CLJ3_6026	NreA-like protein
NA	CLJ1_5843	CLJ3_6025	Inner membrane protein
NA	CLJ1_5844	CLJ3_6024	probable membrane protein YPO3302
NA	CLJ1_5845	CLJ3_6023	hypothetical protein
NA	CLJ1_5846	CLJ3_6022	hypothetical protein
NA	CLJ1_5847	CLJ3_6021	Chromate transport protein ChrA
NA	CLJ1_5848	CLJ3_6020	Chromate resistance protein ChrB
NA	CLJ1_5849	CLJ3_6019	Phage integrase family protein
NA	CLJ1_5851	CLJ3_6018	RuBisCO operon transcriptional regulator CbbR
NA	CLJ1_5852	CLJ3_6017	Phosphonate dehydrogenase (EC 1.20.1.1) (NAD-dependent phosphite dehydrogenase)
NA	CLJ1_5853	CLJ3_6016	Phosphonate ABC transporter permease protein phnE (TC 3.A.1.9.1)
NA	CLJ1_5854	CLJ3_6015	Phosphonate ABC transporter phosphate-binding periplasmic component (TC 3.A.1.9.1)
NA	CLJ1_5855	CLJ3_6014	Phosphonate ABC transporter ATP-binding protein (TC 3.A.1.9.1)
ns	CLJ1_5856	CLJ3_6013	FIG002188: hypothetical protein

ns	CLJ1_5857	CLJ3_6012	FIG067310: hypothetical protein
NA	CLJ1_5858	CLJ3_6011	hypothetical protein
ns	CLJ1_5859	CLJ3_6010	Long-chain-fatty-acid--CoA ligase (EC 6.2.1.3)
NA	CLJ1_5860	gap	Enoyl-CoA hydratase (EC 4.2.1.17)
NA	CLJ1_5861	ns	Mobile element protein
NA	CLJ1_5862	ns	Mobile element protein
NA	CLJ1_5863	gap	transcriptional regulator, TetR family
NA	CLJ1_5864	CLJ3_5612	Error-prone, lesion bypass DNA polymerase V (UmuC)
NA	CLJ1_5866	ns	Mobile element protein
NA	CLJ1_5867	CLJ3_5613	hypothetical protein
NA	CLJ1_5868	CLJ3_5614	Mobile element protein
NA	CLJ1_5869	CLJ3_5615	hypothetical protein
NA	CLJ1_5870	CLJ3_5616	hypothetical protein
NA	CLJ1_5871	CLJ3_5617	hypothetical protein

NA: no orthologous gene is found in the corresponding genome, ns: orthologous gene(s) is/are found in other location(s) in the corresponding genome, gap: the gene location corresponds to a gap between contigs in the corresponding genome.

Table S6. BLASTN search result of CLJ-ISL3 (2985 bp) against the ISfinder database with E-value of 0.

Sequences producing significant alignments	IS Family	Origin	E-value	Identity (%)	Query length (bp)	Query coverage (%)	Gaps (%)
ISPst2	ISL3	*Pseudomonas stutzeri*	0,00	99,30	2984	100	0,43
ISPpu12	ISL3	*Pseudomonas putida*	0,00	99,27	3372	43	0,00
IS1396	ISL3	*Serratia marcescens*	0,00	99,27	1771	41	0,00
ISPst9	ISL3	*Pseudomonas stutzeri*	0,00	90,40	2472	41	0,00

Table S11. CLJ phage-related regions.

**CLJ1 locus**	**Region**	**Number of genes**	**Corresponding PA7 locus**	**Mobility gene present**
**0462-0497**	RGP66 (including CLJ-SR1)	36	PSPA7_0678-0716	Integrase
**0535-0557**	RGP3	23	PSPA7_0754-0775	None
**2607-2624**	RGP26 (including CLJ-SR6)	18	PSPA7_2648-2661	Integrase
**4295-4314**	Including CLJ-SR11	20	PSPA7_4602-4606	None
**4778-4791**	RGP78	14	PSPA7_5040-5080	Integrase
**5192-5203**	CLJ-SR13	12	NA	Integrase
**5758-5774**	Including RGP6	17	PSPA7_4699-4703	Integrase
**6156-6161**	CLJ-SR15	6	NA	None

NA: not applicable
